# Closantel Retinal Toxicity: Case Report and Literature Review

**DOI:** 10.1155/2021/4832965

**Published:** 2021-05-21

**Authors:** Sepideh Ghods, Elias Khalili Pour, Hamid Riazi-Esfahani, Hooshang Faghihi, Bahman Inanloo

**Affiliations:** Retina Ward, Farabi Eye Hospital, Tehran University of Medical Sciences, Tehran, Iran

## Abstract

A 57-year-old shepherd was referred with a 2-week history of decreased visual acuity in both eyes. Optical coherence tomography (OCT) (Heidelberg Engineering GmbH, Heidelberg, Germany) revealed diffuse outer nuclear layer hyperreflectivity and indistinguishable external limiting membrane and ellipsoid zone. The patient announced to us that he took two 500 mg of closantel tablets (15.15 mg/kg) three days before the initiation of visual problems for sore throat as an antibiotic. Electroretinography displayed severely attenuated responses in both eyes. We decided to admit the patient with the presumed diagnosis of closantel retinal toxicity and treated him with intravenous methylprednisolone 1 g per day and intravenous erythropoietin 10000 IU twice a day, and reevaluation of the patient proved no change in his visual acuity on the third day of admission. Closantel is a veterinary drug with serious side effects in the human retina and central nervous system even in previously reported doses. Public awareness and appropriate drug labeling about its side effects could prevent accidental toxicity. OCT is a noninvasive and rapid diagnostic modality that should be done in suspected toxic retinopathy.

## 1. Case Presentation

**Figure 1:**
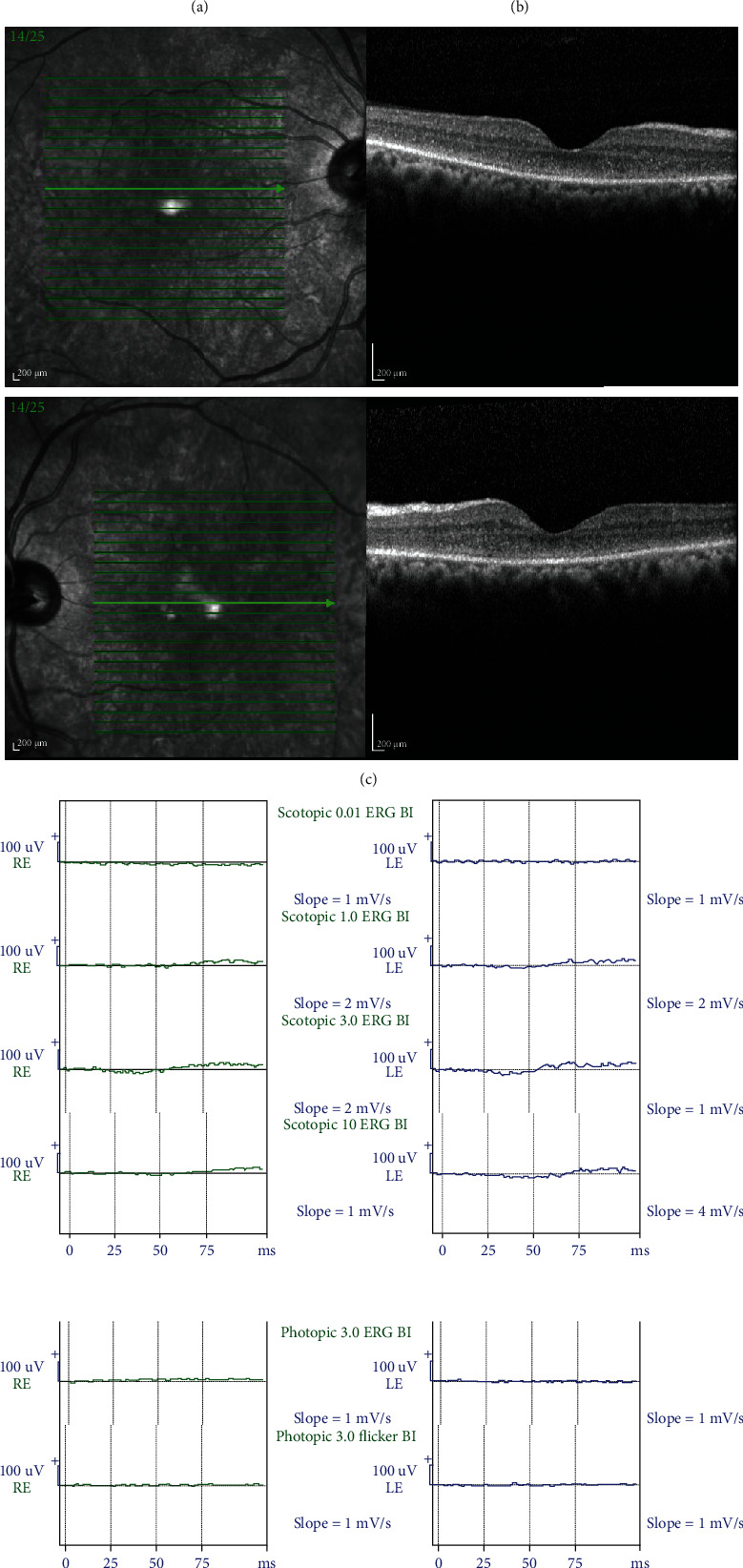
(a) The fundus photograph of both eyes shows a small area of myelinated axons on the inferior margin of the right optic nerve head and very mild peripapillary whitening and nerve fiber layer swelling more prominent in the right eye. (b) Fundus autofluorescence images show a hypoautofluorescent area corresponding to the small area of myelinated axons on the inferior margin of the right optic nerve head and the normal appearance of the left eye. (c) Macular optical coherence tomography (OCT) revealed diffuse outer nuclear layer hyperreflectivity and indistinguishable external limiting membrane and ellipsoid. (d) Electroretinography displayed severely attenuated responses in both eyes.

A 57-year-old shepherd was referred to the retina clinic of Farabi Eye Hospital complaining about 2-week sudden vision loss in both eyes. He weighed about 66 kg. His visual acuity was count fingers at 50 cm and 1 m for the right and left eye, respectively. His past medical history was unremarkable, and he denied any recent self-medication. On slit-lamp examination, the anterior segment was normal except for mild symmetric nuclear sclerosis and middilated and poorly reacting pupils. The fundus exam showed a small area of myelinated axons on the right optic nerve head and very mild peripapillary whitening and nerve fiber layer swelling more prominent in the right eye (Figure 1(a)). Fundus autofluorescence and infrared reflectance (Heidelberg Engineering GmbH, Heidelberg, Germany) images were normal (Figure 1(b)). Optical coherence tomography (OCT) (Heidelberg Engineering GmbH, Heidelberg, Germany) revealed diffuse outer nuclear layer hyperreflectivity and indistinguishable external limiting membrane and ellipsoid zone (Figure 1(c)). Systemic laboratory tests and brain MRI were performed before his referral. Laboratory documents were unremarkable except for the liver enzyme test twice the normal range. T2-weighted brain MRI without gadolinium showed multiple bright foci at peri- and paraventricular, centrum semiovale, and junctional areas (not showed) that were diagnosed as a microvascular disease by a radiologist.

Based on our previous experience on closantel retinal toxicity and its typical OCT features, we returned to the patient's history and asked about inadvertent veterinary drug use. The patient announced to us that he took two 500 mg of closantel (15.15/kg) tablets 3 days before the initiation of visual problems for sore throat as an antibiotic. Electroretinography displayed severely attenuated responses in both eyes (Figure 1(d)). As we came across some plasma exchange beneficial reports in literature [1], we referred the patient to toxicologists but they were reluctant for performing the procedure, as there was a low chance of recovery. Consequently, we decided to admit the patient and treated him with intravenous methylprednisolone 1 g per day and intravenous erythropoietin 10000 IU twice a day and reevaluation of the patient proved no change in his visual acuity on the third day of admission.

## 2. Discussion

Closantel is an old veterinary antihelmetic drug used broadly for the treatment of Fasciola and Haemonchus infestation [2]. It is a halogenated salicylanilide that acts on the energy metabolism pathway by blocking oxidative phosphorylation [3]. There are several reports of the toxic effect of closantel on the central nervous system, optic nerve, and retinal tissues in animals [3–5]. However, reports in humans are limited and mainly was associated with visual manifestations. Therefore, closantel is contraindicated in humans and should be used with caution in milk-producing animals. It has been claimed that a low dose of closantel is safe in humans [6], but our case clarified that it could be devastating in doses as low as 1000 mg.

Previous studies on the animal brain and ocular tissues explained pathologic changes. Gill et al. showed severe edema in the myelinated intracranial optic nerve and optic tract and small scattered foci of myelinic edema in the brain stem and cerebellar peduncles of goats and sheep. They observed retrograde axonal degeneration because of myelinic edema and its compressive effect at the intracanalicular portion of the optic nerve [5]. Studies on retinal tissues demonstrated acute and severe outer retinal cells, particularly photoreceptor necrosis [2].

In 1993, ‘t Hoen and Hodgkin published a series of blindness in 11 women following mistaken treatment for gynecological problems [7]. Badran et al. reported 3 cases of severe visual impairment following accidental closantel ingestion [8]. There is another paper from Morocco about blindness in a 5-year-old girl after 8 days of closantel ingestion with partial visual recovery that occurred after medical treatment by vitamin K, vitamin B12, and glucocorticoid [9]. Koziolek et al. reported a case of visual loss after 3 days of closantel intake. They performed plasma exchange 4 days after the last dose, and the patient experienced significant visual recovery [1]. Previous studies in animals showed that closantel reaches its peak plasma level after 8-48 hours of oral administration and its half-life in plasma was 2-3 weeks. As closantel has a high serum protein binding capacity and low metabolism rate, plasma exchange in the early phase after ingestion can effectively remove it from circulation [1, 10]. Tabatabaei et al. treated a case of blindness due to closantel toxicity with intravenous steroid and erythropoietin which did not affect visual prognosis [11]. Recently, Khalili and Zareei reported a complete recovery of vision and electroretinography parameters of a patient with retinal closantel toxicity by single intravenous injection of methylprednisolone acetate 250 mg. Unlike our patient, their case was referred early and steroid therapy commenced on the 5^th^ day after intoxication [12]. Since there was 2 weeks of delay in referral and diagnosis of our patients, toxicology specialists refuse to do plasma exchange. Administration of high doses of intravenous methylprednisolone and erythropoietin was also ineffective, so we consider these medical management options futile in closantel toxicity.

In our patient, OCT revealed diffuse outer nuclear layer hyperreflectivity and indistinguishable external limiting membrane and ellipsoid zone. Tabatabaei et al. reported very similar findings of OCT in a closantel toxicity case [11]. OCT image is an inexpensive, widely available, and easy to interpret modality. We believe OCT could be a key diagnostic imaging for early detection of closantel toxicity. Additionally, ERG showed severely reduced and flat responses in both photopic and scotopic conditions, which indicates severe photoreceptor loss.

Para- and periventricular bright spots on brain MRI in our patient might be due to the toxic effect of closantel on central neural tissues of the visual pathway. Visual evoked responses could show reduced response in this situation, but because severe retinal impairment would affect VER, we did not accomplish this modality. A mild increase in serum liver enzymes has also been reported after higher doses of closantel, but this was observed in our case with far lower doses of the drug.

In a recent study, Asoklis et al. assessed late ocular changes in 5 patients with inadvertent use of at least three tablets of 500 mg closantel. Visual acuity changes were partly reversible, but visual-field defects deteriorated with time, and changes in the fundus, like retinal thinning, were seen. They concluded that closantel has a long-term detrimental impact on the retina, with no discernible recovery after 22 years [13].

## 3. Conclusion

Closantel is a veterinary drug with serious side effects in the human retina and central nervous system even in previously reported doses. Public awareness and appropriate drug labeling about its side effects could prevent accidental toxicity. OCT is a noninvasive and rapid diagnostic modality that should be done in suspected toxic retinopathy. Since early treatment by plasma exchange could remove the drug from circulation and lessen its damages, timely diagnosis is very mandatory.
